# Phosphatidylserine externalization as immune checkpoint in cancer

**DOI:** 10.1007/s00424-024-02948-7

**Published:** 2024-04-04

**Authors:** Ivan-Maximiliano Kur, Andreas Weigert

**Affiliations:** 1 Faculty of Medicine, Institute of Biochemistry I, Goethe-University Frankfurt, Theodor-Stern-Kai 7, 60590 Frankfurt, Germany; 2 Frankfurt Cancer Institute, Goethe-University Frankfurt, 60596 Frankfurt, Germany; 3 German Cancer Consortium (DKTK), Partner Site, Frankfurt, Germany; 4 Cardiopulmonary Institute (CPI), 60590 Frankfurt, Germany

**Keywords:** Cancer, Inflammation, Phospholipids, Macrophages, Immune checkpoint

## Abstract

Cancer is the second leading cause of mortality worldwide. Despite recent advances in cancer treatment including immunotherapy with immune checkpoint inhibitors, new unconventional biomarkers and targets for the detection, prognosis, and treatment of cancer are still in high demand. Tumor cells are characterized by mutations that allow their unlimited growth, program their local microenvironment to support tumor growth, and spread towards distant sites. While a major focus has been on altered tumor genomes and proteomes, crucial signaling molecules such as lipids have been underappreciated. One of these molecules is the membrane phospholipid phosphatidylserine (PS) that is usually found at cytosolic surfaces of cellular membranes but can be rapidly and massively shuttled to the extracellular leaflet of the plasma membrane during apoptosis to serve as a limiting factor for immune responses. These immunosuppressive interactions are exploited by tumor cells to evade the immune system. In this review, we describe mechanisms of immune regulation in tumors, discuss if PS may constitute an inhibitory immune checkpoint, and describe current and future strategies for targeting PS to reactivate the tumor-associated immune system.

## Introduction

Cancer is the collective term for a large number of diseases that are caused by somatic mutations, leading to unchecked expansion of the cells in which the mutations occurred. This expansion eventually disturbs the physiology of the organ in which the transformation occurred. Moreover, during cancer development, which in humans can take decades, transformed cell can acquire the ability to leave the organ of origin and colonize distant sites to form metastases. Distant metastasis is a major reason why, despite considerable progress in cancer therapy, cancer remains a leading cause of non-natural death globally [17]. While the goal of cancer therapy is to remove the transformed cells from the body, it has become apparent that focusing therapeutic efforts merely on the cancer cells alone may not be sufficient. Rather, the complex interplay of the cancer cells with their cellular and molecular neighborhood, the tumor microenvironment (TME), needs to be considered and ideally be targeted to aid in removing the transformed cells. This is of particular importance because cancer cells acquire the ability to subvert their local microenvironment from a naturally tumor-suppressive into a tumor-supporting state [14, 25, 30].

The TME is composed of a specific extracellular matrix, various gradients of nutrients and gaseous molecules, and different cell types such as vascular and lymphatic endothelial cells, mural cells, fibroblasts, and immune cells [33]. A major limiting factor of tumor growth is the immune system, which is able to sense neo-antigens and stress-induced molecules in transformed cells, which under normal circumstances leads to eradication of the developing cancer [7, 71, 83]. The occurrence of clinically detectable tumors therefore indicates that immune control has failed. Indeed, many advanced tumors are characterized by a tumor-promoting rather than protective immune response. Identifying the factors that shape the formation of such a detrimental immune environment with the aim to interfere with their action to re-activate the immune system against a tumor has consequentially become a major research goal. Hereby, tumors appear to exploit physiological mechanisms that have evolved to limit immune reactions during disturbed tissue homeostasis or to switch off immunity once the trigger of disturbed homeostasis has been removed. These central mechanisms that decide if an immune reaction is initiated, persists, or is terminated are called immune checkpoints [73]. They are commonly viewed as intercellular receptor-ligand interactions. In this review, we discuss under which conditions PS may serve as an immune checkpoint in cancer and discuss the underlying mechanisms and strategies to target PS to overcome tumor-associated immune suppression.

## Immune checkpoints in cancer

A considerable part of the attention related to research for cancer therapy within the last two decades has been directed to the field of cancer immunotherapy. This is due to several key findings including the prognostic relevance of the immune response in patients, the success of cellular immunotherapy, and immune checkpoint blockade (ICB). The cellular composition of the tumor-associated immune system and the functional profile of these cells have emerged as a powerful predictor of prognosis for cancer patients [8]. The presence of immune cells with an activation profile reminiscent of anti-microbial or anti-viral defense responses such as CD8 + T cells, T helper 1 (TH1)-polarized CD4 + T cells, memory T cells, NK cells, γδ T cells, B cells, and activated myeloid cells in the TME is associated with a favorable outcome, while immunosuppressive myeloid cells and regulatory T cells (Treg) that are able to limit the anti-tumor properties of the former cells predict poor prognosis in most cancers [8, 116]. An immunosuppressive TME is not only prognostically relevant, but also a major hurdle for immunotherapy approaches that aim at supplying protective immune cells to cancer patients [55]. The mechanisms that render the TME immunosuppressive are highly diverse in nature [93], but disrupting inhibitory immune checkpoints has taken center stage due to its clinical efficacy [106, 107].

ICB is an approach to prevent immunosuppressive molecular interactions mediated by inhibitory immune checkpoints by the administration of neutralizing antibodies. Immune checkpoints are defined as the engagement of a receptor expressed on immune cells by its ligands, whose expression is often more widespread. Receptor engagement either enhances or suppresses effector function of the cells expressing the receptor to constitute either co-stimulatory or co-inhibitory immune checkpoints [107]. Physiologically, co-inhibitory immune checkpoints are engaged to prevent autoimmunity and to modulate the duration and extent of immune responses [73]. These inhibitory checkpoints are usually not expressed or expressed at low levels on resting cells but are upregulated upon activation to provide negative feedback signaling opportunities. A major focus has been on such immune checkpoints expressed by T cells that modulate the second signal of T cell activation following the recognition of antigens presented by major histocompatibility molecules via their specific T cell receptor [85]. The most understood co-inhibitory immune checkpoint molecules are cytotoxic T-lymphocyte-associated protein 4 (CTLA-4) and programmed death 1 (PD-1), both regulating distinct non-redundant inhibitory signaling pathways [106]. CTLA-4 is upregulated on T cells after antigen recognition and competes with the co-stimulatory molecule CD28 for its ligands, the B7 family molecules CD80 and CD86 [45], which are expressed on antigen-presenting cells following their activation. CTLA-4 negatively regulates T cell activation to fine-tune or terminate immune reactions by a remarkable plethora of mechanisms, including the removal of the CD28 ligands from the immunological synapse by trans-endocytosis [76, 106]. Allison and colleagues demonstrated that the application of CTLA-4 blocking antibodies resulted in complete tumor rejection and long-lasting immunity in mice due to a potentiated anti-tumor immune response [49]. PD-1 is also upregulated on T cells after antigen-dependent activation but is expressed by other cells such as B cells and macrophages as well [1]. PD-1 interaction with its ligands PD-L1 and PD-L2, which in comparison to CD28/CTLA-4 ligands are widely expressed on a variety of cell types [69], predominantly interferes negatively with T cell receptor-dependent signaling [23]. Honjo and colleagues demonstrated that PD-1-deficient mice were prone to develop autoimmunity indicating PD-1 as a crucial negative regulator of lymphocyte signaling [34, 70]. These remarkable pre-clinical findings sparked further research efforts, culminating in the first immune checkpoint disrupting antibodies entering clinical trials. Remarkable clinical response rates for CTLA-4 and PD-1 blocking antibodies were observed in melanoma patients [29, 48, 81], which was followed by a number of further clinical studies in other cancer entities [106]. Most importantly, ICB yielded long-term survival benefits in some cancer patients. Interfering with the PD-1/PD-L1 immune checkpoint showed superior clinical benefit compared to CTLA-4 blockade, which might be due to the less restricted expression pattern of PD-1 and its ligands, particularly PD-L1 in tumor cells.

**Fig. 1:**
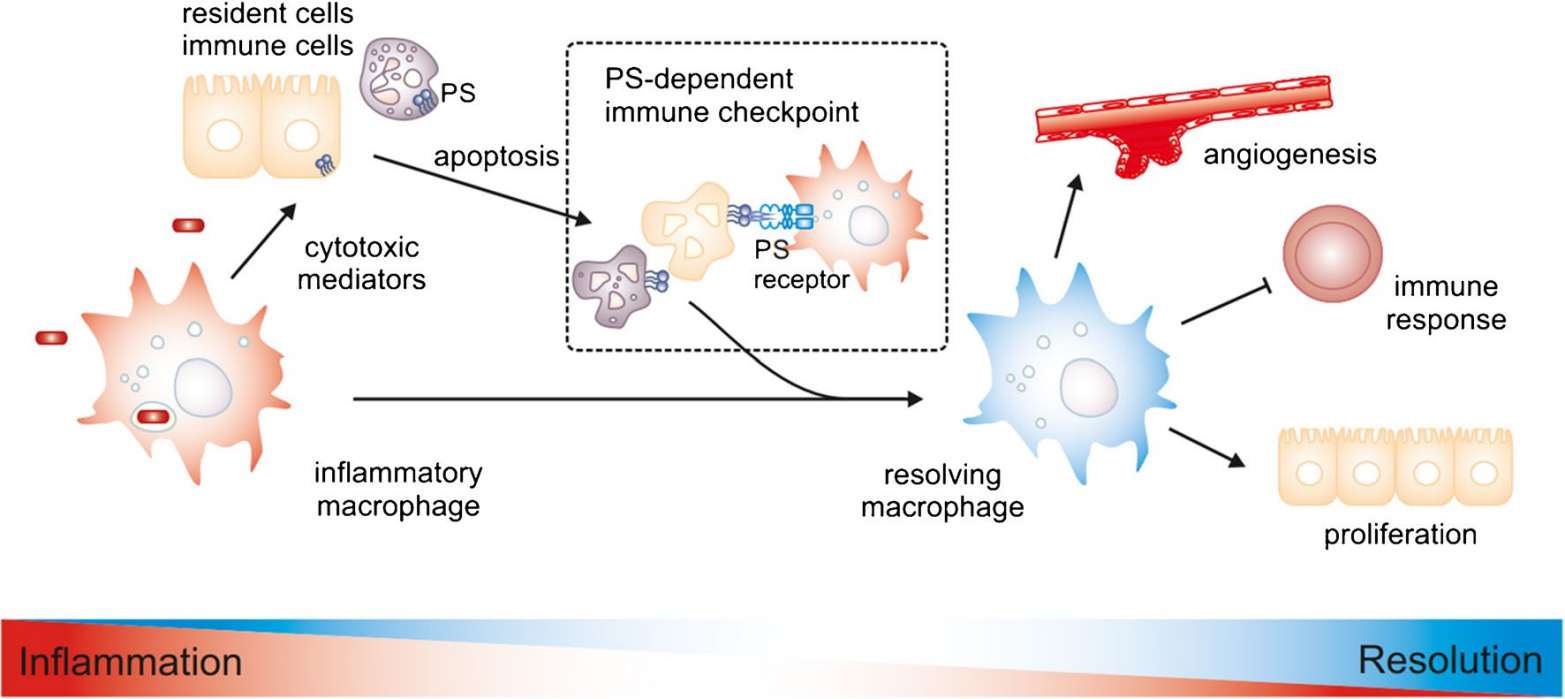
The PS immune checkpoint. PS is externalized under conditions of tissue damage, e.g., during inflammation. Externalized PS is recognized by macrophages, which are then programmed towards resolution of inflammation and tissue repair. Thus, PS is a signal to limit and terminate immune reactions and can therefore be considered a non-conventional inhibitory immune checkpoint

Despite this remarkable success story, ICB only works in a subgroup of patients, and induced resistance to ICB has emerged from follow-up studies [86]. However, the predictive power of the immune response holds even in cancer entities where current ICB strategies show low efficacy. This indicates that new targets to activate the immune system against cancer are needed. Hereby, other immune cells, apart from those that interact primarily or directly against the tumor, should be considered. For instance, tumor-associated macrophages are key contributors to shaping the tumor-promoting TME and are able to remove tumor cells via phagocytosis [122]. The signals that educate macrophages to support tumor growth are manifold, including the recognition of dying tumor cells [24, 108]. Physiological cell death and particularly the recognition of dying cell-derived molecules can serve as a signal limiting inflammatory macrophage activation under physiological conditions, and may therefore be viewed as a myeloid cell-targeted immune checkpoint [109]. Hereby, PS is a central signal to recognize dying cells, induce their removal by efferocytosis, and induce repair pathways in macrophages (Fig. 1).

## Phosphatidylserine metabolism

PS is an essential anionic glycerophospholipid that comprises about 3–10% of total phospholipids in mammalian cells under physiological conditions, which is the scenario the following description is focusing on. Glycerophospholipids are amphiphilic molecules that are the major constituents of all cellular membranes. They are usually composed of a central glycerol moiety with two hydrophobic ester-linked fatty acyl groups in the sn1 and sn2 position and various different hydrophilic headgroups linked to the glycerol via a phosphate molecule in the sn3 position, which makes them amphiphilic. Lysophospholipids with a fatty acid only in the sn1 or sn2 position and glycerophospholipids with ether-linked fatty alcohols also exist, albeit in lower quantities. Importantly, the distribution of glycerophospholipids in membrane bilayers, generally being arranged in a manner that the hydrophobic tails are directed towards the center of the bilayer and the hydrophilic heads are oriented towards intracellular or extracellular fluids, is not uniform. Rather, a discrete distribution of glycerophospholipids with different headgroups is essential for eukaryotic cell physiology [27, 118]. PS is highly enriched in the cytosolic layer of cellular membranes, even though it can be found at the luminal side of the membrane bilayer in cellular organelles such as the endoplasmic reticulum, mitochondria, and the Golgi complex [18, 97]. The asymmetric distribution of PS in cellular membranes is established and maintained by ATP-dependent flippases that catalyze the localization of PS to the cytosolic leaflet of the plasma membrane [68]. This resulting state appears to be highly stable [43], but specific PS scramblases exist that are able to rapidly interfere with the asymmetrical distribution of PS in an ATP-independent manner, resulting in its exposure at the extracellular leaflet of the plasma membrane [68].

PS is synthesized by two distinct phosphatidylserine synthase (PTDSS) enzymes, PTDSS1 and PTDSS2 [104]. Both enzymes are localized in the endoplasmic reticulum in mitochondria-associated membranes [103], where they catalyze a base-exchange reaction replacing the polar headgroup of phosphatidylcholine (PC) or phosphatidylethanolamine (PE) with serine. PTDSS1 and PTDSS2 differ in their expression profiles in distinct cell types [3, 5, 92] and in their substrate specificity. While PTDSS2 utilizes exclusively PE as substrate, PTDSS1 appears to be more promiscuous, accepting both PC and PE [42]. Particularly, PTDSS1 may be involved in generating ether-linked PS species [84], even though PTDSS2 can also accept ether-linked PE as substrate with relatively low efficacy [41]. Ether-linked PS are probably rare in cells even though data are limited. In the CNS, 13% and 0.3% 1-O-alkenyl hydrocarbon chains that result from linking fatty alcohols to glycerol were found in PS in white matter and gray matter, respectively, while PE contained 47% and 21% 1-O-alkenyl hydrocarbon chains in white and gray matter [39]. Thus, ether-linked PS levels may vary between 0.01 and 1.3% among glycerophospholipids in tissues. Despite these substrate preferences, PTDSS isoforms are able to compensate for each other. Even though mice lacking *Ptdss1* showed 85% reduced serine-exchange capability, the overall PS content was unaltered due to the compensatory action of PTDSS2. A similar phenomenon was reported vice-versa in *Ptdss2*^−/−^ mice, while double deletion of PTDSS1 and PTDSS2 was lethal [3]. Degradation of PS occurs in mitochondria by phosphatidylserine decarboxylase (PISD), which decarboxylates the serine headgroup in PS to ethanolamine thereby generating PE. Transport of PS from mitochondria-associated membranes to other cellular membranes, including the plasma membrane, has been reviewed in detail [37, 42] and mainly involves vesicular transport and a minor contribution of soluble lipid transfer proteins.

Localization of PS both at the cytosolic and the extracellular leaflet of the plasma membrane mediates crucial cellular functions, which are largely defined by its anionic headgroup. Hereby, the net negative charge results from the combination of the serine and phosphate moieties. Cytosolically oriented PS provides a major proportion of the negative charge density of the plasma membrane’s inner leaflet, allowing a number of proteins to interact with PS. This occurs either in a non-specific manner due to the presence of polycationic regions in their primary structure, which are found for instance in Ras- and Rho-GTPases and the protein kinase Src, or in a specific manner via C2 domains which bind Ca^2^^+^ to link proteins such as protein kinase C or phosphoinositide-3-kinase to PS and, consequently, the plasma membrane [37, 59]. Localization of these proteins to the plasma membrane is essential for their functionality, which includes triggering major survival pathways in cells. This is in line with findings that higher eukaryotic cells lacking the machinery to synthesize PS do not survive [3]. These intracellular signaling properties of PS are active during steady-state conditions. In contrast, PS is externalized or otherwise visible to the extracellular milieu when cellular homeostasis is disturbed. Here, it may under some circumstances act similar to an inhibitory immune checkpoint.

## PS and its receptors in immunity

Intracellular PS is part of platforms for triggering crucial signaling modules for immune cell activation such as protein kinase C [2] or pathways downstream of antigen recognition by T cell receptors [113]. Its exposure at the extracellular leaflet of the plasma membrane occurs in a variety of conditions, including immune cell activation, platelet activation, and cell death, and usually requires suppression of flippase activity, induction of scramblase activity, or both [6, 37]. Hereby, the expression of PS at the outer plasma membrane leaflet of viable cells is generally a transient event that is required during developmental processes such as myoblast or osteoclast fusion. PS exposure on activated immune cells is known for a long time, but the physiological relevance remains unknown. Activated neutrophils transiently expose PS, which is not related to cell death [19]. In mast cells, PS exposure is induced after stimulation of the IgE receptor or experimental elevation of cytosolic Ca^2+^, and degranulation occurs, indicating that PS exposure could be associated with exocytosis [60]. Indeed, there is further evidence supporting a role in exocytosis, since PS exposure is observed at exocytic sites of activated chromaffin cells [72]. T cells may also express PS in the outer leaflet of the plasma membrane without undergoing apoptosis [87]. Interestingly, non-apoptotic PS exposure on T cells promotes infection with human immunodeficiency virus (HIV)-1 and is triggered by the viral envelope glycoprotein of HIV-1 itself interacting with CD4. PS exposure during HIV-1 infection depends on Ca^2+^-dependent activation of TMEM16F scramblase, and blocking PS exposure protects from infection [120]. Also during platelet activation, Ca^2+^-dependent activation of TMEM16F scramblase and probably inactivation of P4 ATPase family flippases result in PS exposure, serving as a platform for serine protease activation during coagulation [68]. Hereby, serine proteases in the coagulation cascade bind to PS at the platelet surface via their Ca^2+^-containing gamma-carboxyglutamic (Gla) domain. Production of active thrombin during that process can in turn propagate inflammation [10].

In contrast to these mechanisms that may promote immune reactions, PS exposed on the surface of apoptotic cells avoids or even actively prevents and resolves inflammation [6]. PS is exposed on the surface of apoptotic cells due to caspase-dependent inactivation of P4 ATPase family flippases and activation of XK-related protein family scramblases, particularly Xkr8 [68]. PS exposure is just one of the consequences of apoptotic cell death. Downstream effects of caspase activation to execute apoptosis comprise loss of organelle integrity, DNA fragmentation, and plasma membrane blebbing with preserved plasma membrane integrity to generate apoptotic bodies. These are then removed by macrophages before secondary necrosis can occur. Apoptosis occurs very frequently during homeostatic conditions and is therefore non-inflammatory and non-immunogenic to maintain tolerance and to prevent a systemic auto-immune response [61, 108, 110]. It is important to note that in the context of cell death, PS exposure does not selectively occur during apoptosis. Various modes of cell death including apoptosis, necrosis, and necroptosis are characterized by the exposure of PS towards the extracellular milieu. However, the specific mechanisms and kinetics appear to be different, including the loss of overall plasma membrane integrity and accessibility of the extracellular milieu to its inner leaflet during necrosis, ion fluctuations (Ca^2+^ and Cl^−^), and caspase activity, which affect flippase and scramblase activity [88]. In each of these cases, PS serves as a signal to remove the cellular debris, a so-called eat-me signal. However, particularly during apoptosis, its properties surpass serving as a first signal for debris removal. Recognition of PS by a large array of receptors is one of the reasons why apoptosis is non-immunogenic, inducing active signaling events to counteract inflammatory signaling and to activate tolerogenic pathways [6]. This property of PS may even be used to actively trigger the resolution of inflammation as PS-containing liposomes acting on local macrophages in inflammation models restricted inflammatory leukocyte recruitment, while PC-containing liposomes did not [90].

Sensing of PS in the extracellular space requires dedicated receptors. There are several PS receptors that may act redundantly, but also in a context-specific manner to allow tailored reactions to dying cells [61] (Table 1). PS receptors include brain angiogenesis inhibitor 1 (BAI1) [46], stabilin-1/2, αVβ3-5 integrins [26, 78], CD300 family proteins [12, 65, 89], T cell/transmembrane, immunoglobulin, and mucin (TIM) family proteins, and TYRO3, AXL, and MER (TAM) receptor tyrosine kinases. BAI1, stabilin-1/2, and the TIM family proteins directly bind to PS, while TAM and integrin receptors indirectly bind to PS via bridging molecules [74]. TAM receptors bind PS via the Gla domain-containing proteins growth arrest-specific 6 (GAS6) and protein S (PROS1), and integrin receptors bind to PS via MFGE8 [6, 61].

**Table 1:** Expression and function of PS receptors

PS receptor	Cell type expressing receptors	Primary functions	References
TIM-1	CD4 + T cells, Th2 cells, NKT cells, regulatory B cells, mast cells,	T cell activation and cytokine production	[20, 99, 117]
TIM-3	Th1 cells, Th2 cells, Th17 cells, regulatory T cells, CD8 + T cells, NKT cells, B cells, NK cells, mast cells, dendritic cells, monocytes, macrophage subpopulations	Anti-inflammation, immune tolerance, promotion of tumor growth	[20, 63, 111]
TIM-4	Dendritic cells, macrophages	Inflammatory responses, maintenance of tolerance	[20, 57, 112]
BAI-1	Macrophages	Phagosome formation and transport	[46, 74]
Stabilin-1	Macrophages	Healing and repair, promotion of tumor growth	[11, 74]
Stabilin-2	Macrophages	TGF-β production	[11, 40, 75]
α_V_β3-5 integrins*	Macrophages	Macrophage activation, efferocytosis promotion, or inhibition	[26, 78]
CD300 family proteins	Macrophages, NK cells, T cells, B cells, neutrophils, plasmacytoid dendritic cells, mast cells, eosinophils	Positive or negative regulation of efferocytosis dependent on individual receptor	[12, 65, 74, 89]
TYRO3* (via Gas6 and Pros1)	Dendritic cells	Immunosuppression	[54, 82]
AXL* (via Gas6)	Dendritic cells and macrophages	Immunosuppression, tissue repair, resolution of inflammation, macrophage activation, promotion of tumor growth	[54, 74, 95, 119]
MERTK* (via Gas6 and Pros1)	Macrophages, dendritic cells, NK cells, B cells, CD4 + T cells, CD8 + T cells	Immune tolerance, resolution of inflammation, T cell activation, macrophage activation, promotion of tumor growth	[21, 54, 82, 119]

*These receptors do not interact directly with PS but with a soluble bridging molecule that couples to PS

Stabilin-1 and stabilin-2 are type I membrane protein receptors expressed in primary and secondary hematopoietic organs and other immune-related organs such as lymph nodes, spleen, bone marrow, and the liver that upon binding to PS on apoptotic cells initiate phagocytosis [40]. Anti-inflammatory macrophages that are associated with tissue repair have been shown to express stabilin-1 [75]. Stabilin-2 is also expressed in macrophages and participates in TGF-β production, which is an immunosuppressive cytokine produced by efferocytic macrophages that regulates immune cell generation and function [75].

The TIM family consists of type I cell surface glycoproteins, which have been identified as direct PS receptors. TIM-1 is expressed on CD4 T cells, mast cells, and B cells. Interaction of TIM-1 with PS reduces T cell activation and cytokine production [20, 99, 117]. TIM-4 is expressed on dendritic cells and macrophages [112]. Particularly in liver macrophages expressing TIM-4, PS recognition via TIM-4 attenuated NLRP3 inflammasome activation in the context of nonalcoholic fatty liver diseases [57]. TIM-3 is a co-inhibitory receptor that is designated as an immune checkpoint and is expressed on a variety of cells including diverse T cell subsets, dendritic cells, and macrophages [63]. Several ligands for TIM-3 beside PS were identified, explaining its broad anti-inflammatory relevance in transplant tolerance, autoimmunity, viral infections, and cancer [111].

The TAM family proteins are linked to PS via the bridging molecules GAS6 and PROS1, where the amino terminus binds to PS in a Ca^2+^-dependent manner while the carboxy terminus binds to the individual TAM receptor. The three TAM receptors share a similar structural organization but are expressed on different cells, show different modes of interaction with GAS6 and PROS1, and may be linked to different signaling pathways [54, 82, 96]. For instance, mice lacking TYRO3 show neurological disorders, mice lacking AXL show vascular problems, while MERTK-deficient mice present autoimmunity issues. However, all three appear to contribute to efferocytosis and regulate inflammation [15]. MERTK, the most studied TAM receptor regarding immune response, is mainly expressed by macrophages, even though expression on other cells such as dendritic cells, natural killer cells, B cells, and T cells has been noted [21]. MERTK signaling in macrophages plays a role in both inflammatory disease and cancer and has generally been connected with the resolution of inflammation and tissue repair. In contrast, triggering AXL-dependent signaling on macrophages can have detrimental and even diametrically opposed functional outcomes compared to MERTK [15, 119].

The data summarized above indicate that PS recognition on the surface of apoptotic cells is usually anti-inflammatory, but context-specific responses are enabled by receptor diversity. It is important to highlight that not all externalized PS causes the equal responses. Externalized PS on viable cells or on activated platelets does not classically result in their removal by macrophages, and PS externalized in the context of necrosis does at least not strongly limit the inflammatory properties of necrotic cells [88]. The downstream effects of recognition of externalized PS probably depend on concentration, topology, or its clustering capacity, as demonstrated for its interaction with AXL [62]. Future studies will need to address the composition of the lipid and protein environment of PS, saturation of fatty acids and fatty alcohols in PS, and oxidative status, e.g., by mass spectrometry to assess under which conditions the presentation of PS to the extracellular space leads to immunosuppressive or stimulatory outcomes. However, it appears reasonable to assume that tumors may utilize the undeniable immunosuppressive potential of PS to evade the immune system.

## Role of PS in cancer

Assuming that tumors may exploit the immunosuppressive nature of PS would indicate that a significant degree of cell death happens in tumors. This assumption is not trivial as protection from cell death is a hallmark of cancer. While this is certainly true in a subpopulation of cancer cells, the proliferative index in tumors usually comes along with a higher degree of cell death compared to the parent tissue [108, 110]. Thus, tumors as such can use the non-immunogenic properties of PS to hinder their eradication by the immune system by sacrificing a proportion of their own. While this occurs under steady-state conditions, the immunosuppressive properties of PS may become even more relevant upon cancer treatment. The main therapy regimens used in clinical practice including surgery, chemo-, and radiotherapy induce either cell death in tumor cells themselves or may cause death of local tissue cells or rapidly dividing cells systemically, which will cause a significant degree of immunosuppressive PS exposure [38]. Also, killing tumor cells by current immunotherapies including ICB will induce significant PS exposure on the dying tumor cells. Consequently, targeting PS and its receptors in cancer might produce synergistic effects when combined with current therapy regimens.

Indeed, several PS receptors have been connected to tumor immunity. For instance, mice with a deletion of stabilin-1 in macrophages showed reduced tumor growth compared to control mice [75]. The TAM family receptors are often classified as proto-oncogenic receptors in tumor cells themselves and have been described in different types of cancers, playing a role in proliferation, migration, survival, and chemo-resistance properties. High expression of these receptors is related with poor prognosis and cell aggressiveness. This may, among others, also stem from their role in recognizing PS expressed on (dying) tumor cells [66]. Indeed, the removal of apoptotic cells by MERTK-expressing macrophages induced an anti-inflammatory phenotype and promoted metastasis in breast cancer [91]. MERTK blockade by antibodies led to an anti-tumor response characterized by type 1 interferon production, with additive effects after combination with ICB [123]. Mice harboring MERTK-deficient myeloid cells showed tumor resistance, slower tumor growth, and enhanced expression of inflammatory cytokines, accompanied by higher presence of CD8 T cells. Depletion of CD8 T cells restored tumor growth in this model. These effects were observed in melanoma and mammary carcinoma models, but MERTK blockade caused an increase in the growth of colorectal cancer models [13]. Such opposite effects in different cancer models may be related to a heterogeneous expression of PS in the tumor environments and/or the presence of alternative PS receptors. However, in a model of acute lymphoblastic leukemia (ALL), the inhibition of MERTK decreased tumor burden and prolonged survival. MERTK inhibition decreased the expression of PD-L1 and PD-L2 on monocytes/macrophages and decreased PD-1 expression in T cells, leading to increased CD4 + and CD8 + T cell activation [50]. Also, AXL/GAS6 signaling was actively exploited by leukemic cells to generate a suppressive TME by driving macrophages towards a tumor-promoting phenotype. Depletion of both AXL or GAS6 in macrophages stimulated NK cell- and T cell-dependent immunity against the leukemia cells and sensitized to ICB [95]. These data summarized above suggest that targeting the TAM receptor family holds the potential to potentiate anti-tumor effects of ICB in different types of cancer.

Other efforts to utilize PS dysregulation in the TME have focused on targeting PS itself. Some blocking strategies consist of the administration of PS ligands such as Annexin A5 (AnxA5) or others, which may slow tumor progression and increase the immunogenicity of tumor cells [56]. In addition to merely blocking PS recognition with AnxA5, fusing AnxA5 with other peptides or proteins has been investigated. For instance, bacterial L-methionase, which catalyzes the degradation of the essential amino acid L-methionine, was linked to AnxA5 to target it specifically to tumor cells. This led to reduced methionine availability and furthermore induced the conversion of selenomethionine into toxic methylselenol, thereby killing tumor cells and inhibiting tumor growth [102]. Moreover, tagging tumor antigens to AnxA5 significantly enhanced its immunogenicity and anti-tumor efficacy when administered after chemotherapy, which as expected increased PS exposure. The efficacy of re-activating the immune system with AnxA5-dependent delivery of tumor antigens was further enhanced by ICB [36, 52]. Besides AnxA5, antibodies against PS were tested in tumor models. When the PS-binding antibody 2aG4 was used in a model of prostate cancer in combination with chemotherapy, the immune profile of the TME shifted to stimulate anti-tumor responses, which was characterized by an increased abundance of pro-inflammatory macrophages and mature dendritic cells suppressing progression of the tumors [114]. Another preclinical PS targeting antibody, mch1N11, improved the anti-tumor immune response of radiotherapy in a melanoma model, which was further improved when ICB was added. The combination generated a potent cytotoxic T cell response even against contra-laterally placed non-irradiated tumors [9]. Lactadherin, another multifunctional glycoprotein, binds PS-enriched cell surfaces in a Ca^2+^-independent manner. This interaction is crucial for regulation of blood coagulation, but it was also observed that lactadherin affects the reprogramming of pro-tumoral anti-inflammatory macrophages, reducing glioma growth [35], probably by preventing the recognition of PS.

In addition to targeting PS receptors or PS exposure to the extracellular milieu, recent findings have suggested that reducing PS synthesis may be beneficial in cancer. PTDSS1 expression was frequently upregulated in different cancer types and was associated with poor survival [84]. At least in murine breast tumor cells, increased PTDSS1 expression resulted in increased PS levels [84]. Deletion of PTDSS1 in breast cancer cells reduced PS levels and tumor growth, which was associated with reduced abundance of macrophages, due to reduced proliferation, in tumors. This phenotype was mimicked in mice lacking MERTK in macrophages, suggesting that PS/MERTK signaling was needed for macrophage proliferation and subsequent support of tumor growth [84]. Hereby, PTDSS1 deletion mainly affected the production of ether-linked PS species but reduced PS exposure upon apoptosis. How ether-linked PS regulates PS exposure during apoptosis remains unclear. Interestingly, increased PS exposure upon PTDSS1 overexpression has been reported previously [100], and early reports suggested that PS exposed during apoptosis was derived from a pool of newly synthesized PS, but a dependency on PTDSS1 or PTDSS2 was not found [105]. Thus, PTDSS1 dependency may be exclusive to tumor cells. This may be of particular relevance given the fact that PTDSS2 expression is lost in a variety of cancer types [115]. Disrupting PTDSS1 activity may therefore specifically affect tumor cells rather than untransformed cells that still express PTDSS2 to produce PS. Accordingly, potent and selective PTDSS1 inhibitors were developed, and their application in PTDSS2-deficient tumor models resulted in tumor regression [115]. This inhibition caused the activation of the endoplasmic reticulum stress response, which mediated cell death and anti-tumor immunity activation, specifically stimulating dendritic cells. Importantly, increased immunogenicity translated into protection against PTDSS2-WT tumor cells in the surrounding, indicating the induction of overall protective immunity [115]. Thus, targeting PTDSS1 appears to interfere with the immunosuppressive TME by reducing the exposure of PS in the outer membrane leaflet, which importantly may not affect efferocytosis per se [6, 84]. The use of pharmacological compounds targeting PTDSS1 may be of interest for tumor therapy. A potential synergy with ICB would also need to be explored, based on the reports above indicating such a synergy between inhibiting PS receptors and ICB.

Another potential role played by PS during tumor immune responses is being a tumor antigen itself, which would be highly expressed due to increased PTDSS1-dependent production in tumors. The inherent diversity of lipids makes them excellent candidates as antigens [64]. Lipid antigens are presented on the surface of antigen-presenting cells via CD1 molecules and are sensed by natural killer T cells (NKT cells), a heterogeneous population of lymphocytes [101]. Among NKT cells, tumor-suppressive type I NKT and tumor-promoting type II NKT cells have been identified [44, 94]. Indeed, lyso-PS species can be recognized by type II NKT cells [84]. Whether and how PS-reactive type II NKT cells affect tumor immunity remains to be determined. Interestingly, CD1d-bound PS may be recognized by PS receptors as well, adding a potential new twist to the immunoregulatory potential of PS [47].

## Clinical implications and strategies to utilize PS exposure for cancer therapy

**Fig. 2:**
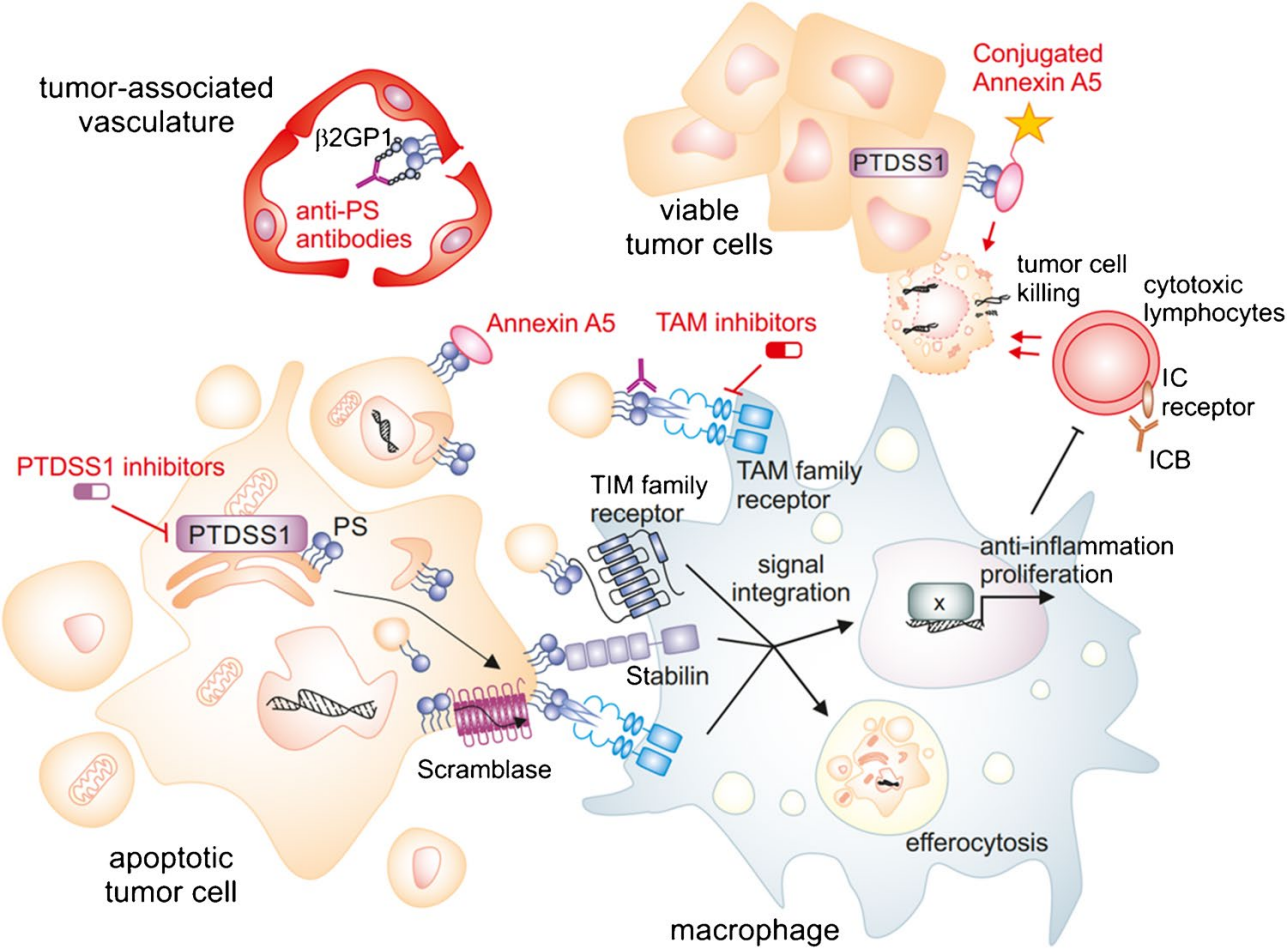
Preclinical and clinical strategies to target phosphatidylserine (PS) in cancer. PS in tumor cells is produced particularly via PS synthase 1 (PTDSS1). It is exposed on the tumor-associated vasculature, living tumor cells, and particularly on dying tumor cells via scramblases. PS is recognized by macrophages, but also other cells, via a divergent set of PS receptors including TAM family receptors, TIM family receptors, and stabilins. PS serves as a signal to remove cellular debris by efferocytosis and induces anti-inflammatory macrophage activation and proliferation. This interaction promotes tumor progression among others by limiting the activity of cytotoxic lymphocytes. PS recognition by macrophages can be interrupted by PS neutralizing antibodies, Annexin A5, and similar PS binding proteins and small molecule inhibitors of PS receptors. Reactivation of cytotoxic lymphocytes by these approaches synergizes with immune checkpoint blockade (ICB). Clinically tested PS antibodies require β2-glycoprotein 1 (β2GP1) to bind to PS and appear to act predominantly at the tumor-associated vasculature. Annexin A5 and similar PS binding proteins can also be linked to imaging or cytotoxic agents or tumor antigens to aid in tumor cell detection or killing. Details can be found in the main text

The evidence summarized above supports the rationale for targeting PS in cancer to overcome immunosuppression [11]. Different pre-clinical and clinical strategies are currently being developed to interfere with PS in cancer (Fig. 2). Besides being a target for immune activation against tumors, PS has been proposed as a marker for clinical monitoring of the efficacy of cytotoxic therapy, i.e., to determine the amount of cell death induced. A number of clinical studies have been performed to investigate the suitability of (99 m)Tc-Annexin A5 as a molecular imaging agent [4]. While data are promising, there appears to be a need for standardization for clinical use. Increased PS exposure on tumor cells and tumor-infiltrating blood vessels is also being tested as a target to direct liposomes specifically towards tumors. Liposomes are lipid vesicles that can be used as drug carriers. Liposomal carriers were synthesized to bind to PS via Saposin C (SapC), a glycoprotein involved in the activation of lysosomal enzymes and ceramide production, which shows a strong affinity for PS. Vesicles containing SapC and dioleylphosphatidylserine (SapC-DOPS) have been used to selectively target different types of cancer in vitro and in vivo while these liposomes do not bind to normal tissue due to low cell surface PS expression. Moreover, they can be used in combination with standard of care therapies [67]. A phase 1 clinical trial with SapC-DOPS is running since 2019, and the last update suggested a tolerable safety profile [80].

The most prominent approach to interfere with PS for immunotherapy is masking externalized PS with an antibody. The monoclonal antibody bavituximab binds to β2-glycoprotein I to subsequently induce its binding to PS with high affinity [58]. Bavituximab has been and is being investigated in clinical trials addressing different types of cancer such as lung, breast, pancreatic, and hepatocellular carcinoma [6]. Unfortunately, a phase III clinical trial for late-stage non-squamous non-small cell lung carcinoma showed no significant improvement of overall survival for patients who received the combination of docetaxel and bavituximab compared to docetaxel alone. However, a potential benefit of combining bavituximab with ICB emerged from this trial [22]. The clinical potential of combining bavituximab with the ICB antibody pembrolizumab is currently explored in phase II trials in gastric and hepatocellular carcinoma [32, 51]. The data reported so far indicate the efficacy of the combination at least in a subgroup of patients that can be identified by specific biomarkers [98]. One major limitation of bavituximab and the preclinical antibodies indicated above may be the requirement of β2GP1 to target PS instead of targeting PS directly. Moreover, these antibodies preferentially bind to the tumor-associated vasculature, which may restrict their potential to reach (dying) tumor cells with externalized PS in order to disrupt their interaction with macrophages and other immune cells [6, 58]. Thus, a major driver of efficacy of these antibodies may be innate immune-driven antibody-dependent cellular cytotoxicity against tumor endothelial cells [77] rather than blocking the immunosuppressive interaction between dying tumor cells and phagocytes. On the other hand, preferential targeting of the tumor-associated vasculature rather than dying tumor cells may avoid undesired side effects. Since PS is a major eat-me signal on dying cells, decreased recognition by phagocytes may lead to an accumulation of apoptotic cells which subsequently can become secondary necrotic, with toxic effects including the tumor lysis syndrome or auto-immune reactions such as the antiphospholipid syndrome [16, 31]. However, as indicated above, decreasing PS levels in tumor cells by deleting PTDSS1 did affect macrophage activation but not efferocytosis [84]. Other eat-me signals together with reduced amounts of exposed PS may suffice for efficient removal of dying cells [61]. Thus, PS-recognizing antibodies with a different mode of action or alternative strategies to interfere with PS recognition should be clinically tested in the future. These would include PTDSS1 inhibitors, particularly in PTDSS2-deficient tumors. Moreover, interfering with PS transport from the endoplasmic reticulum to the plasma membrane may be worth investigating. Generally, mechanisms underlying the synthesis of PS, its transport, distribution, and recycling, need to be studied in more depth to identify new and hopefully selective targets to decrease or increase PS bioavailability depending on the type of therapy needed and the type of cell involved. Of course, the broader the biological function of the target, the more the likelihood of severe side effects increases given the important role of PS as scaffold for intracellular signaling pathways and the fact that billions of cells are cleared daily by efferocytosis.

## Conclusions and outlook

The evidence summarized above supports an important role for PS as an immunomodulatory signal that can be clinically targeted in cancer, even though optimized strategies need to be developed. The outcome of clinical trials of combining bavituximab with ICB in different cancers will provide further insights into the clinical potential of PS-targeting agents. However, alternative approaches to interfere with externalized PS cancer need to be studied as well. These include molecules derived from AnxA5 and alternative agents such as GlaS, a protein derived from the Gla domain of PROS1. Such agents could be used not only to mask PS but also to deliver drugs specifically to the TME [28].

Extracellularly approachable PS probably cannot be considered a prototypical immune checkpoint, even though its upregulation on the extracellular dying cells occurs during an overshooting immune reaction with collateral damage, and is able to limit the ongoing immune reaction to avoid further damage (Fig. 2). PS is upregulated also on living cells and during immune cell activation, where it may promote rather than restrict inflammation. Moreover, some PS receptors such as AXL may under specific circumstances promote detrimental immune reactions. However, PS can serve as a prototypical example for molecular interactions that are frequently overlooked in cancer, i.e., interactions of lipids with other signaling molecules. Considerable efforts have been and are being undertaken to acquire genomic and proteomic information of cancer patients with the idea to identify druggable alterations in these molecules. The overall metabolic consequences of tumor-associated mutations are still largely unexplored, including changes in the lipidome [53]. The few studies that have addressed the tumor lipidome have found remarkable alterations in the composition of lipids forming biological membranes. These alterations affect parameters such as membrane fluidity, which determines mechanic properties of cells, cellular signaling, e.g., due to an altered formation of membrane microdomains, drug uptake, and interaction of cancer cells with the TME [79, 121]. Future systematic efforts are needed to study the cancer lipidome and its impact on the TME. Recent advances in spatial lipidomics will be instrumental towards the latter. This notion also applies to studying the biology of PS per se, particularly the different modes of PS externalization and its recognition. It is still unclear how much PS is actually needed to serve as an eat-me signal if there is a hierarchy of receptors with different affinity and avidity to specific PS species including ether-linked PS, local concentrations, arrangement, or distribution. Increasing sensitivity and development of standards and protocols for unbiased and targeted lipid mass spectrometry, as well as tracing studies using high resolution microscopy, will be helpful to determine these parameters.

## Data Availability

No datasets were generated or analysed during the current study.
